# A novel enhanced FBR and low SAR MIMO antenna with limited ground plane for wearable devices

**DOI:** 10.1038/s41598-025-04234-7

**Published:** 2025-06-04

**Authors:** S. Sesha Vidhya, D. Rajesh Kumar, R. Sri Roshini, Shweta Vincent

**Affiliations:** 1 RMK College of Engineering and Technology, Chennai, India; 2 Department of Electronics and Communication Engineering, Vel Tech Rangarajan Dr.Sagunthala R&D Institute of Science and Technology, Chennai, India; 3 Dr.N.G.P. Institute of Technology, Coimbatore, India; 4 Department of Mechatronics, Manipal Institute of Technology, Manipal Academy of Higher Education, Manipal, 576104 India

**Keywords:** Front to back ratio, Specific absorption rate, MIMO, Wearable devices, SDG 3 (Good Health and Well-being), SDG 9 (Industry, Innovation and Infrastructure), SDG 11 (Sustainable Cities and Communities), Engineering, Electrical and electronic engineering

## Abstract

This study explores a novel approach to mitigate the challenge of a small ground plane inducing substantial back lobes in high front-to-back ratio (FBR) microstrip patch antennas. Introducing a combined technique employing rectangular strip and connecting strip methods, this research aims to counteract backward radiation by generating supplementary magnetic currents through an inductive effect. The hybrid method effectively reduces the backward radiation from the original patch. The proposed single antenna element occupies a notably smaller area (approximately 0.029 λ × 0.036 λ, where λ denotes the cutoff wavelength at 5.9 GHz), making it particularly suitable for wearable applications. Additionally, an equivalent circuit model illustrates the coordinated action, offering comprehensive insights into the physical mechanisms. The achieved FBR surpasses directivity by up to 6.03 dB, leading to a diminished specific absorption rate (SAR) when worn while maintaining minimal impact on radiation efficiency due to human presence, a crucial quality for wearable devices.

## Introduction

The increased demand for modern-day wireless communication tools propels the surge in the progress of wearable wireless devices. Wearable tech has faster information exchange capabilities across various sectors, such as the healthcare domain, surveillance, search and rescue, and online games^1–3^. Guaranteeing seamless data transmission through a reliable wireless link is crucial, mandating the inclusion of top-tier antennas within the framework. Embedded antennas within wearables facilitate the wireless transfer of data between devices on the body and external nodes within the BAN, catering to a broad spectrum of applications^3^. Microstrip antennas have been widely explored for their compactness and adaptability in portable communication systems. Sanad^4^ investigated microstrip antennas on extremely small ground planes, highlighting their potential for miniaturization in wireless devices. The study demonstrated that despite the size constraints, these antennas could maintain reasonable performance, making them suitable for modern communication applications. Ensuring uninterrupted data transmission via a dependable wireless connection is critical, necessitating the integration of high-quality antennas within the system. Leveraging contemporary methods in healthcare systems expands the reach of superior healthcare services to a broader populace, reducing risks and eradicating the delays inherent in traditional manual checkups^5^. As per the World Health Organization (WHO) report^6^, changes in climate, lifestyle, and demographics are driving a significant intensification in communicable diseases. Subsequently, harmonizing the proportion of medical staff and experts to the growing number of patients presents challenges. Adopting intelligent healthcare schemes can markedly expand medical services at a regional level^7–9^.Jehangir and Sharawi^10^ introduced a novel single-layer semi-ring slot Yagi-like antenna. Their design achieved a high front-to-back (F/B) ratio, improving directional radiation characteristics while maintaining a compact form factor. This development is particularly beneficial for applications requiring enhanced directivity and reduced interference from unwanted directions.In a subsequent study, Jehangir and Sharawi^11^ extended their work by designing a multi-input multi-output (MIMO) antenna system based on the semi-ring slot Yagi-like configuration. The proposed MIMO system not only retained a high F/B ratio but also demonstrated improved isolation between elements, making it suitable for modern wireless communication systems. Their findings indicated that the antenna system could effectively enhance signal quality and overall system performance.Making good antennas is easy in perfect conditions with no interference. A proper antenna works well in such a setting. To design an efficient antenna, its size should be about half the wavelength. For example, a 1900 MHz GSM antenna needs to be around 8 cm^12^. But in real life, antennas always face interference. So, researchers are working to improve antennas, balancing size and performance for different uses. Wearable antennas are popular because they provide wireless connection in a small and affordable way. But making them work well in different conditions is a challenge. Factors like temperature, sweat, body contact, clothing, and washing can affect performance^12^. Since wearable tech is used in clothes, antennas need to be part of the fabric. These antennas use conductive textiles combined with other materials^13^. There are two main concerns when designing wearable antennas: how they work when bent and how much radiation they release. The antenna must be flexible to move with the body. Since it is close to the body, it must not release too much radiation. To reduce radiation, researchers use special materials like High Impedance Surfaces (HISs), Electromagnetic Band Gap (EBG) structures, and Artificial Magnetic Conductors (AMCs), which are placed behind the antenna^14,15^. Textile antennas have some problems, like changing frequency due to fabric properties. To fix this, they need to work over a wide range of frequencies^16–18^. Making on-body antennas is hard because they must be small, flexible, and still work well without breaking wireless connections. The human body affects how antennas work, which can reduce their efficiency. Special materials can help reduce this problem^19,20^. Researchers have found ways to improve wearable antennas using HISs, which make them work better and reduce radiation. Meta-surface antennas are also being studied^21^. One study used a 3 × 3 AMC surface on vinyl to keep the antenna away from the body. While this design was small, it had weaker signal strength and lower efficiency^22–25^. In^26^, a bio-medical antenna designed for on-body applications was introduced, featuring a low-profile, compact, conformal design and integrating High Impedance Surfaces (HIS). This configuration mimics the characteristics of a Perfect Magnetic Conductor (PMC), a concept not naturally occurring, thereby providing a unique artificial attribute through the antenna with HIS. This device not only imitates but also governs electromagnetic behaviour. As a result, the wearable antenna showcases notably improved performance in terms of efficiency, directivity, SAR, and front-to-back ratio (FBR). Another pioneering method outlined in^27^ entails a planar Ultra-Wideband (UWB) antenna crafted on a felt textile substrate, ideal for on-body antennas. The design targets a reduction in SAR value while enhancing overall performance, achieved by mitigating backside radiation through the utilization of meta-material (MM). Meta-materials (MMs) are engineered designs that offer two unique advantages that are absent in natural materials. Firstly, they adjust the path of EM waves without causing a phase shift, akin to the capabilities of an Artificial Magnetic Conductor (AMC) and EBG^28^.

Researchers combined rigid and flexible substrates to create an antenna with high gain, efficiency, and low SAR^29^. They improved comfort by adding a conductive cloth ground plane to the textile substrate, boosting gain and lowering SAR. Various wearable antennas with strong isolation and wide bandwidth have been well-documented. In^30^, a proximity-fed textile microstrip patch antenna offered enhanced bandwidth and radiation efficiency, a milestone for wearables. Using a proximity feed widened the antenna’s bandwidth and reduced its size, enhancing adaptability for wearables. This aligns with the growth of wireless body area networks (WBAN) in healthcare, patient monitoring, rescue operations, and wearable gaming consoles. Proposed wearable antennas like cavity-backed, microstrip, inverted-F, planar, and vertical monopole antennas have limitations like bandwidth constraints and protrusion. Integrating EBG structures in wearable antennas resolves these issues by isolating them from the body and reducing SAR. Meta surface-based structures, used in various antennas, enhance directivity at specific frequencies. An advancement in^38^ introduced a miniaturized EBG-backed monopole antenna for the MBAN band but lacked comprehensive system-level validation, focusing on design enhancement without broader operational assessment.

Despite significant advancements in wearable antennas, key challenges persist, particularly in ensuring consistent performance in diverse environments, addressing impedance mismatching, and mitigating the high SAR due to close human-body proximity. Existing solutions, including EBG structures and artificial magnetic conductors (AMCs), improve isolation but often compromise directivity, bandwidth, or integration feasibility in wearable applications. Moreover, microstrip patch antennas with high front-to-back (F/B) ratios often suffer from substantial back lobe radiation when used on small ground planes, limiting their efficiency in practical wearable scenarios.

This study proposes a novel hybrid technique combining rectangular strip and connecting strip methods to counteract backward radiation in high F/B ratio microstrip patch antennas. The design effectively minimizes backward radiation by generating supplementary magnetic currents through inductive effects while maintaining a compact size (0.029 λ × 0.036 λ at 5.9 GHz), making it ideal for wearable applications. The proposed approach achieves an F/B ratio improvement of up to 6.03 dB, significantly reducing SAR levels when worn, all while preserving radiation efficiency. Additionally, an equivalent circuit model provides deeper insight into the physical mechanisms, ensuring optimized performance in real-world wearable environments.

### The major significance of the work is as follows

#### Innovative technique

A new method combining rectangular strip and connecting stub approaches mitigates back lobe radiation in microstrip antennas.

#### Controlled radiation

Using an inductive effect, this hybrid technique effectively reduces backward radiation from the original patch.

#### Compact and wearable

The resulting antenna element occupies a small area, ideal for wearable devices at 5.9 GHz.

#### Insights from model

An equivalent circuit model provides in-depth insights into the method’s workings.

#### Improved performance

Achieved FBR surpasses directivity by 6.03 dB, reducing SAR while maintaining radiation efficiency in the presence of the human body.

### The proposed antenna design and configuration

**Fig. 1:**
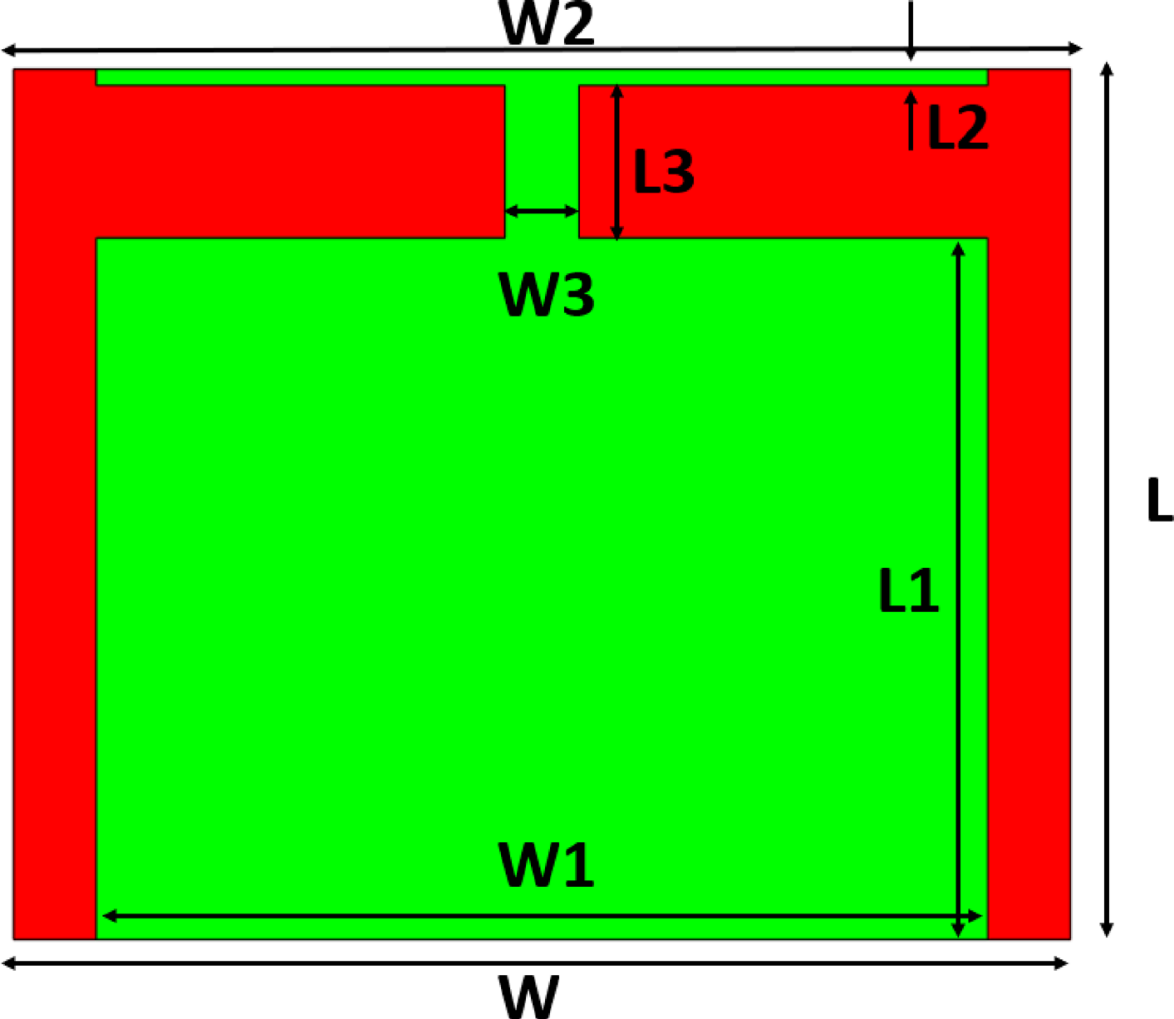
The structure of the proposed enhanced FBR antenna.

**Fig. 2:**
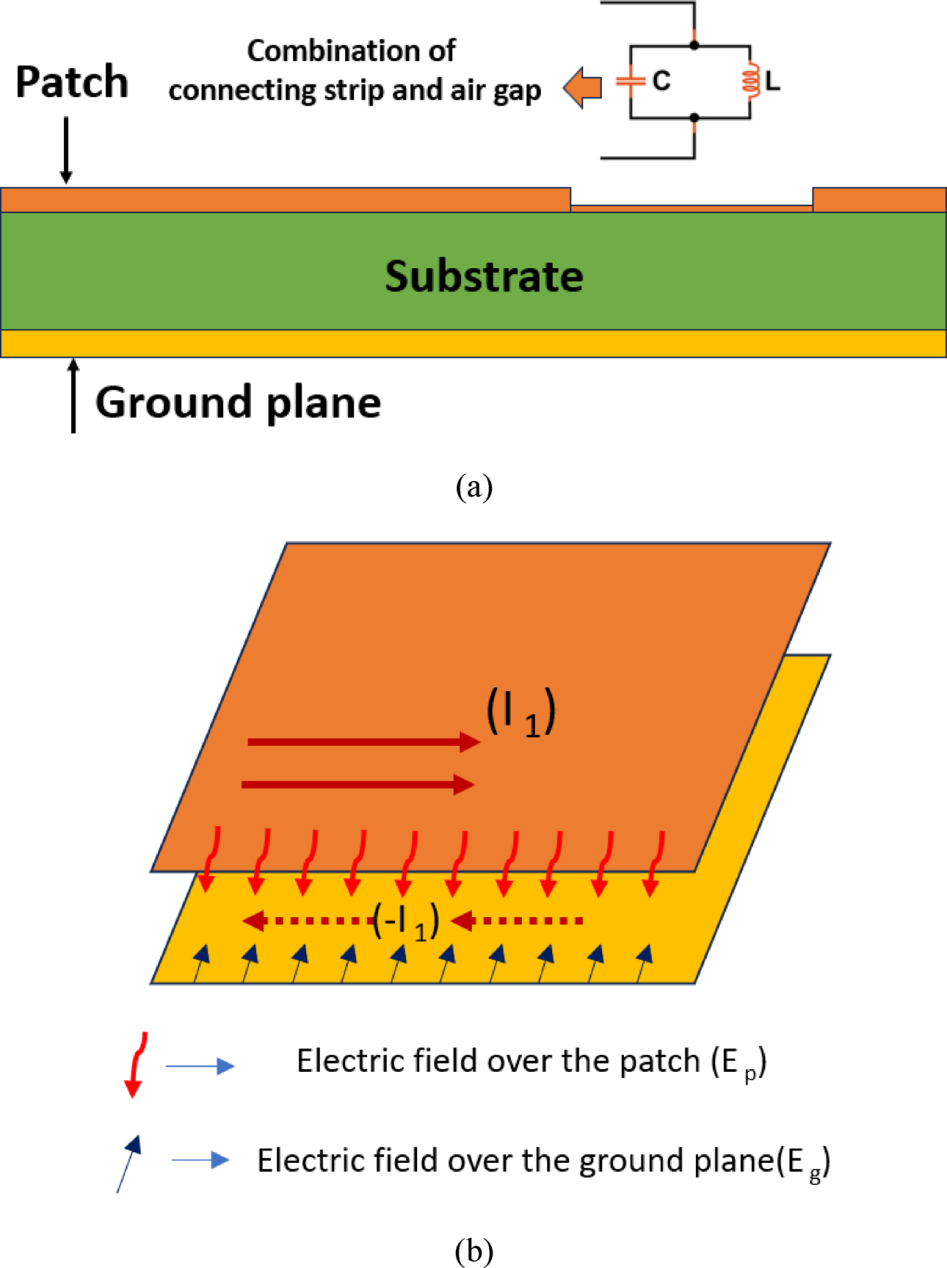
(**a**) Side view and analytical representation of proposed antenna (**b**) Mitigation of back lobe radiation.

In this section, a novel patch antenna design is introduced for minimal backward radiation and compact ground size. It explores operational principles, an FR-4 substrate (1.6 mm height, relative permittivity 4.4), and precise dimensions in Table 1. Figure 1 shows a rectangular patch connected to a small strip through a rectangular stub, while Fig. 2a depicts a side view of the proposed antenna with the full ground plane. Figure 2b illustrates how the connected stub reduces surface current at the ground back, suppressing back lobes and demonstrates the connecting stub’s operation, generating new current pathways through inductance and coupling capacitance. The tangential electric fields at the patch and ground slot, suppressing back lobe radiation through opposite magnetic currents. This approach harnesses lightweight, cost-effective, and compact design advantages for portable devices. The proposed antenna was designed and simulated using CST Microwave Studio 2021.

**Table 1:** Various parametrs of the proposed antenna.

Parameters	L	W	L1	W1	L2	W2	L3	W3
Values (mm)	15.22	18.5	12.2658	15.6	0.287	15.6	2.6672	1.2912

**Fig. 3:**
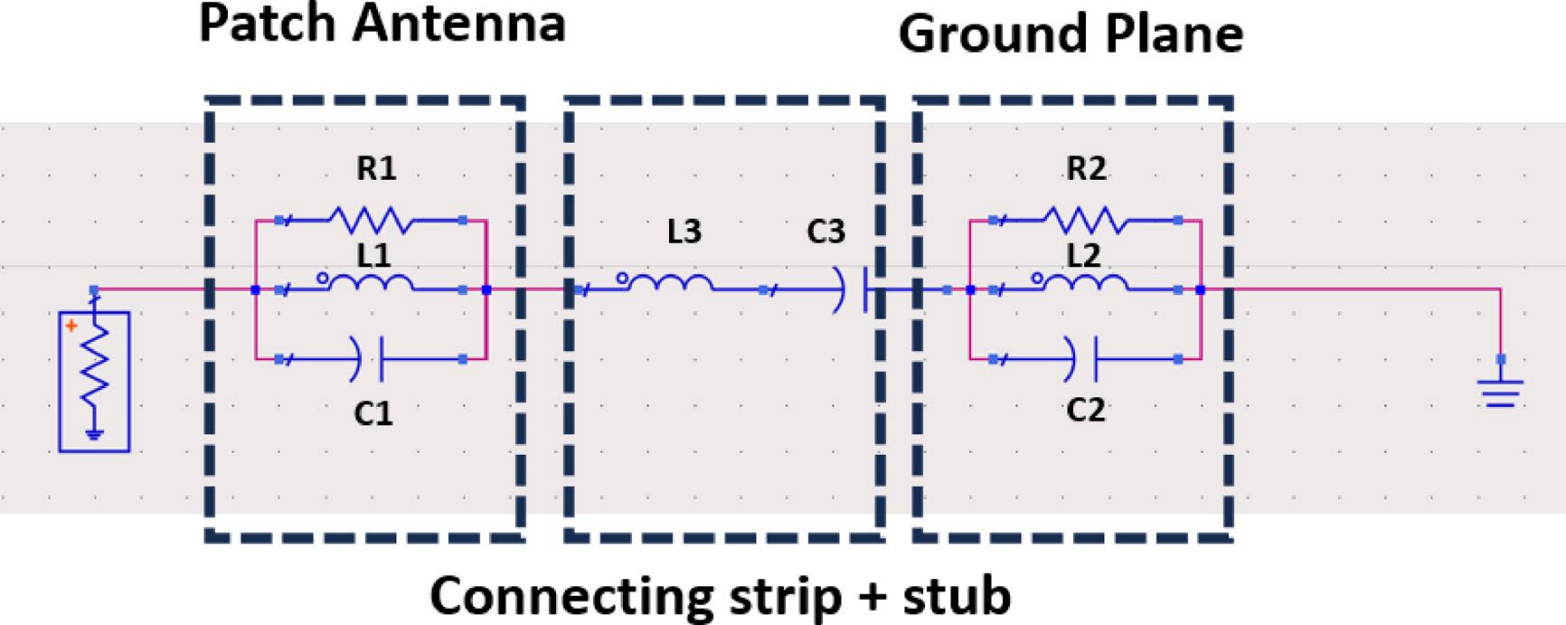
Equivalent circuit model of the designed antenna.

Figure 3 displays the equivalent circuit model, portraying the patch’s radiating edge as a parallel circuit with elements including radiation resistance (R1), inductance (L1), and capacitance (C1). Similarly, the ground slot is represented by parallel components R2, L2, and C2. The interconnected connecting strip and stub structure are illustrated as a series incorporating coupling capacitance (C3) and parasitic inductance (L3). To uphold a consistent voltage (V) across resistor R1, the mathematical expression for the voltage Vs across Rs is determined as follows:

$$\frac{{E}_{s}}{{E}_{r}}=\frac{1}{\left[\left(\frac{1}{{XC}_{1}}+\frac{1}{{XC}_{3}}+{XL}_{3}\right)\left(\frac{1}{{XL}_{2}}+{XC}_{2}\right)+1\right]}$$

**Fig. 4:**
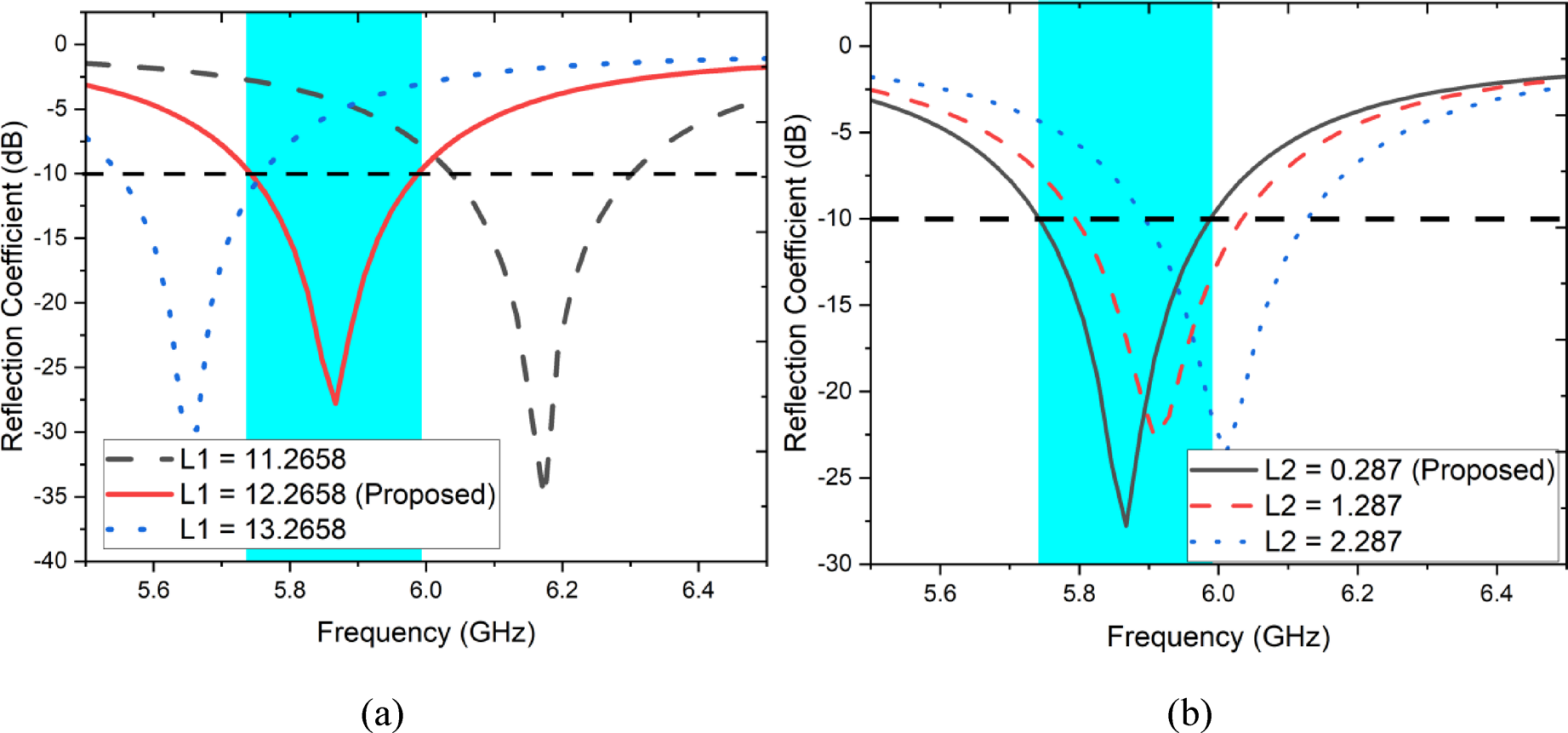
Parametric study when altering (**a**) L1, (**b**) L2.

Parametric analysis has been carried out to investigate the effects of various parameters of the designed antenna. As shown in Fig. 4a,b the influence of parameters L1 (length of the patch) and L2 (Length of the stub) on reflection coefficient have been studied. As shown in Fig. 4a while changing L1 from 11.2658 to 12.2658 mm it is apparently noted that significant shift in frequency of operation. It is obvious that length of the radiating patch affecting frequency of resonance. On the other hand, variations in L2 have also been disturbing the frequency of operation as shown in Fig. 4b.

**Fig. 5:**
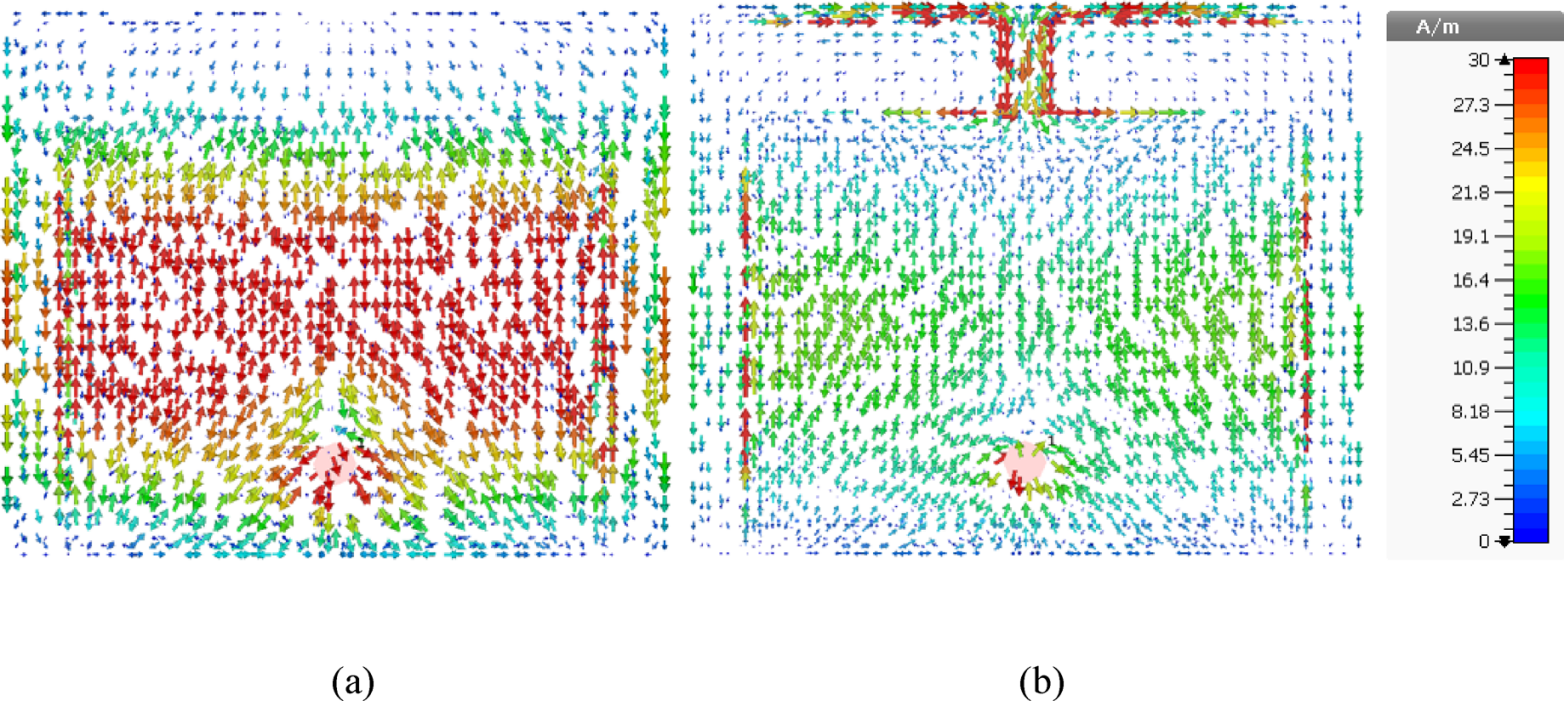
Surface current distribution of the proposed antenna (**a**) conventional patch antenna, (**b**) Proposed antenna.

The ground current distribution of the proposed antenna with and without connecting stub is illustrated in Fig. 5a,b respectively. It is observed that, in the absence of stub and connecting branch peak current is along the centre of the ground plane as in Fig. 5a, whereas when they (stub and connecting branch) are incorporated the peak current has been shifted along the top corner of the ground plane which leading to high front to back ratio (suppression of the back lobes) as shown in Fig. 5b.

The reduction in back lobe radiation significantly impacts the SAR of the proposed patch antenna. This substantial decrease indicates negligible human body absorption from the radiator, rendering the wearable device safer and ensuring steadier antenna performance.

**Fig. 6:**
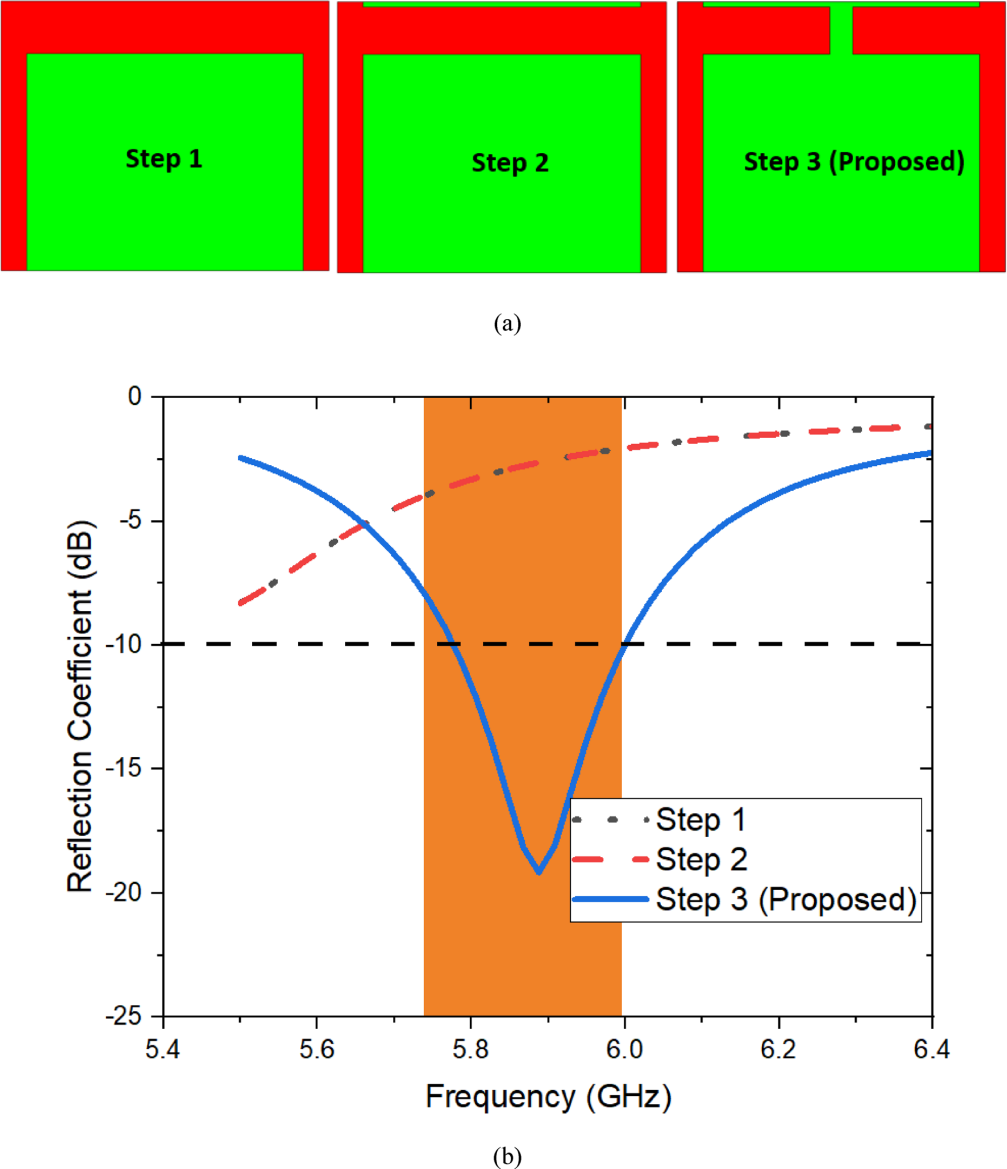
Developing stages of proposed antenna and their (**b**) Reflection coefficient.

The Fig. 6a represents that the evolution stages of the proposed antenna and their corresponding reflection coefficient is illustrated in Fig. 6b. It is clearly observed that the antenna with main patch (Step 1) and stub at the edge of the substrate (Step 2) unable to resonate at the intended frequency of operation from 5.7 to 6 GHz due to lack of current flow over the surface of the radiating patch as well as limited ground plane size. When incorporating both the main patch and corner stub with connecting branch offers the resonance due to the effect of inductance and capacitance.

### MIMO configuration

**Fig. 7:**
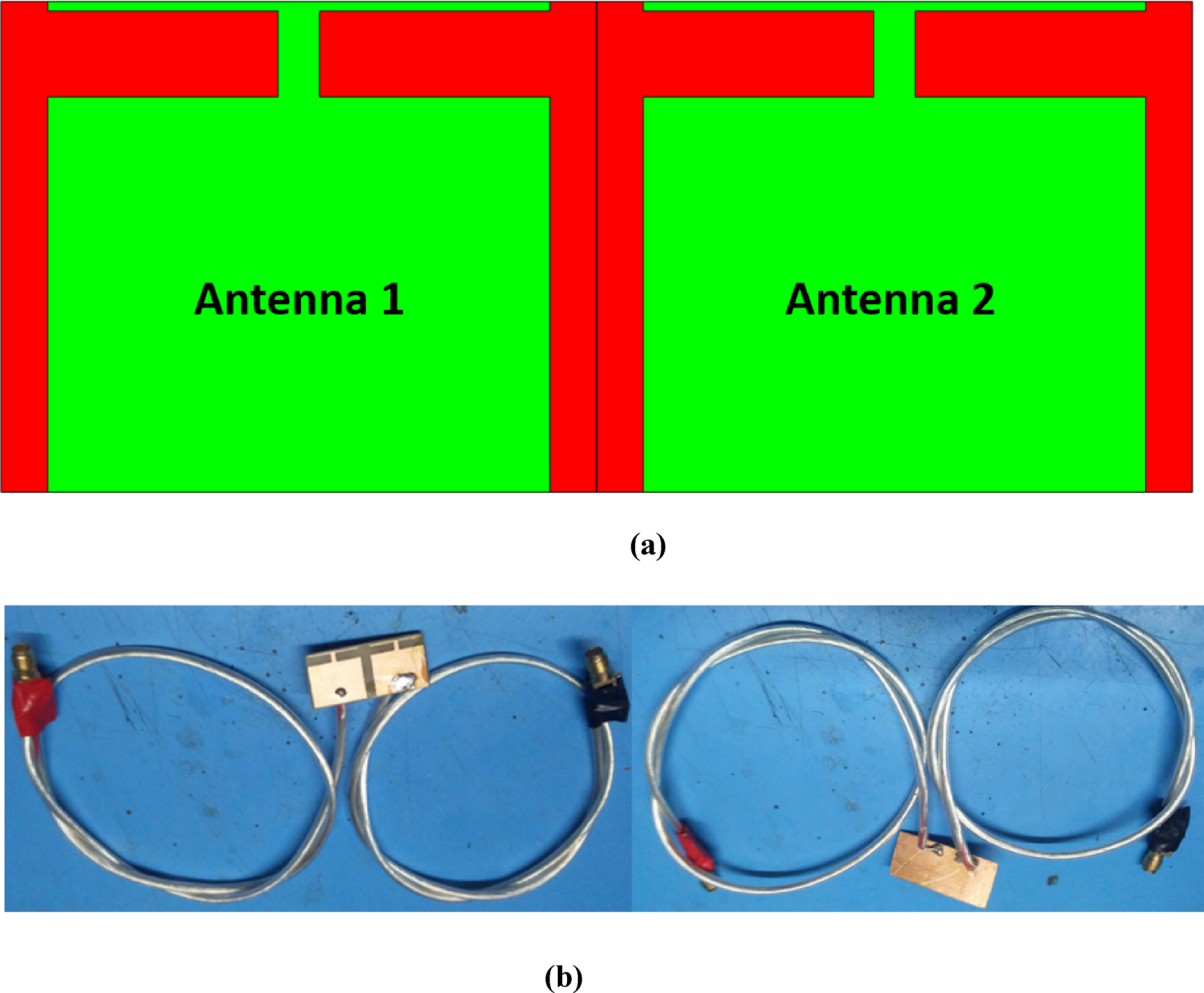
(**a**) Designed MIMO configuration, (**b**) Fabricated protype.

The MIMO configuration of the proposed antenna is illustrated in Fig. 7a. Two identical antennas are placed together with common ground plane. It is important to mention that distance between two radiating element is 2.9 mm, with such minimum distance coupling between two antennas are as low as 12.5 dB. The overall area of the proposed antenna is measured to be 37 mm × 15.22 mm which is significantly smaller for modern day wireless devices.

**Fig. 8:**
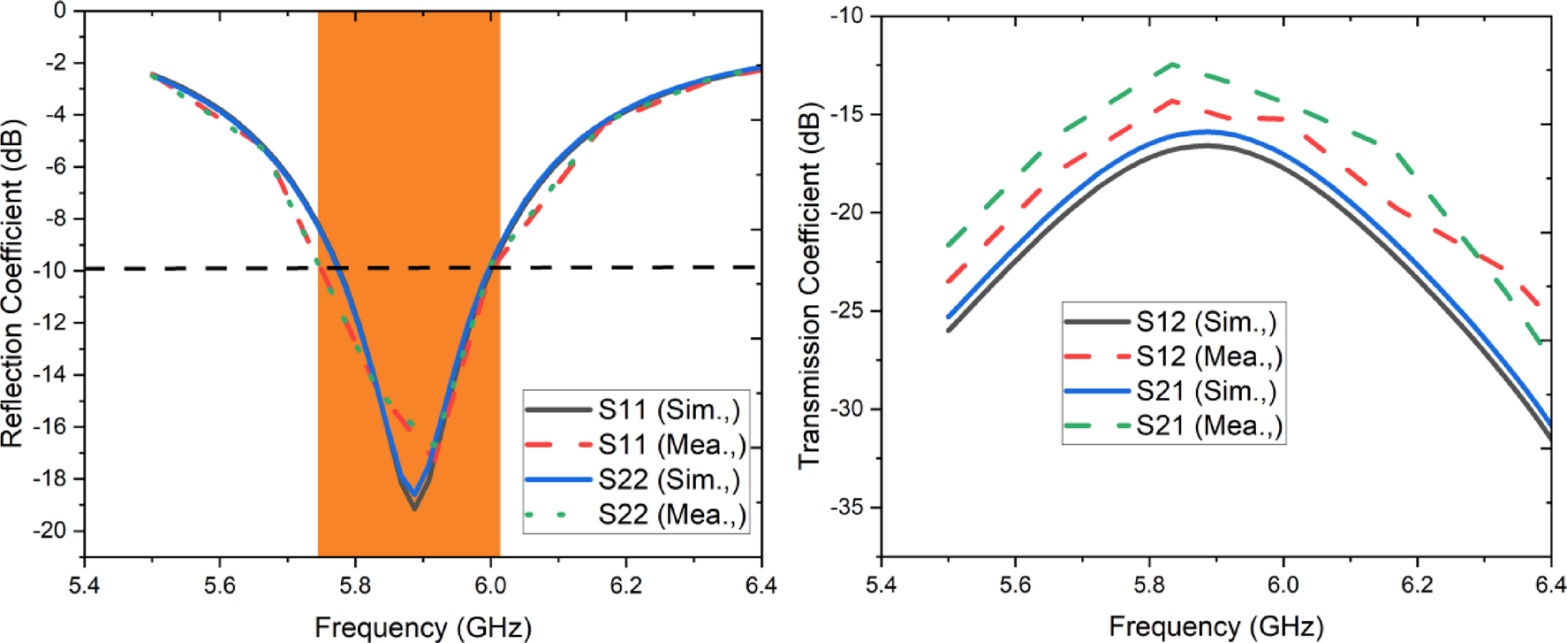
Simulated and measured S-parameters (**a**) Reflection coefficient, (**b**) Transmission coefficient.

The designed MIMO configuration is fabricated and illustrated in Fig. 7b. Each antenna is fed by 50-Ω SMA connector and while taking measurements either of the antenna has been terminated by 50-Ω terminator. The S parameters of the designed antenna is measured using Keysight’s vector network analyzer. The simulated and measured reflection coefficients are depicted in Fig. 8a and it is noticed that both the antennas span between 5.75 and 6.025 GHz over 10 dB impedance bandwidth. The simulated and measured isolation between each element is observed to be lower than 12.5 dB as shown in Fig. 8b.

**Fig. 9:**
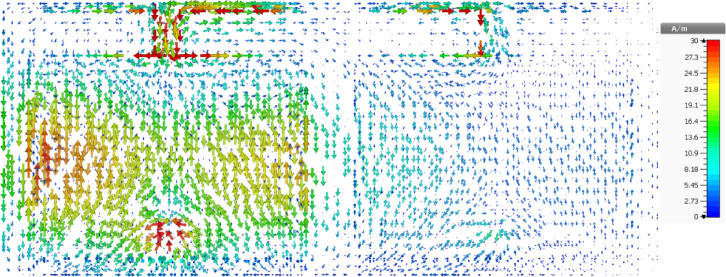
Surface current distribution of the proposed antenna at 5.9 GHz.

**Fig. 10:**
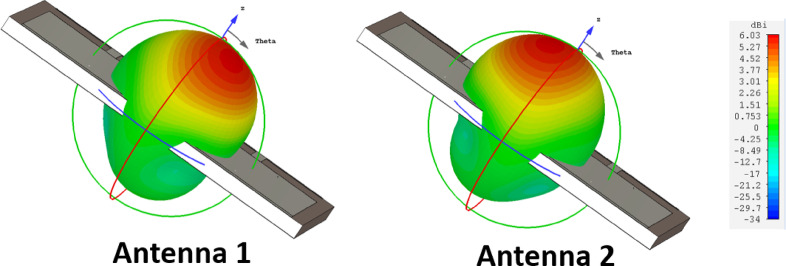
Simulated three-dimensional radiation pattern (**a**) Antenna 1, (**b**) Antenna 2.

The surface current distribution of the designed MIMO antenna when port 1 is excited is illustrated in Fig. 9. It is clearly observed that antenna 2 is completely detached from the current flow in antenna 1, leading to an isolation of better than 12.5 dB. The simulated three-dimensional radiation pattern of antenna 1 and 2 are illustrated in Fig. 10a,b, respectively. As discussed earlier, the peak radiation is observed over a positive z-direction with minimum lobes, proving that the proposed antenna exhibits a substantial front-to-back ratio. Also, it is observed that the designed antenna exhibits considerable directivity, around 6.03 dB in the desired region of operation.

**Fig. 11:**
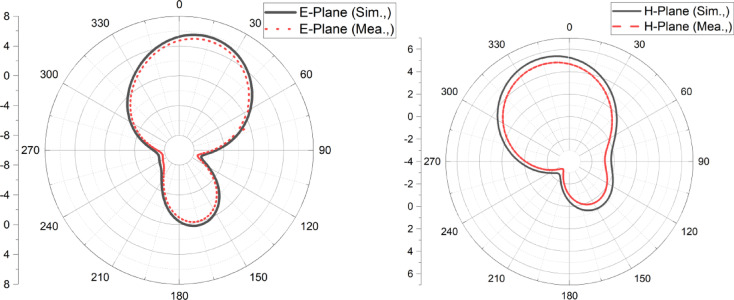
Simulated and measured two-dimensional radiation pattern of Antenna 1 (**a**) E-plane, (**b**) H-Plane.

The simulated and measured E and H plane far-field patterns of the proposed antenna are depicted in Fig. 11a,b. It is noticed that the proposed two-element antenna exhibits a highly unidirectional radiation pattern over the desired band of operation, and the measured patterns are well correlated with simulated patterns, as shown.

### MIMO parameters

**Fig. 12:**
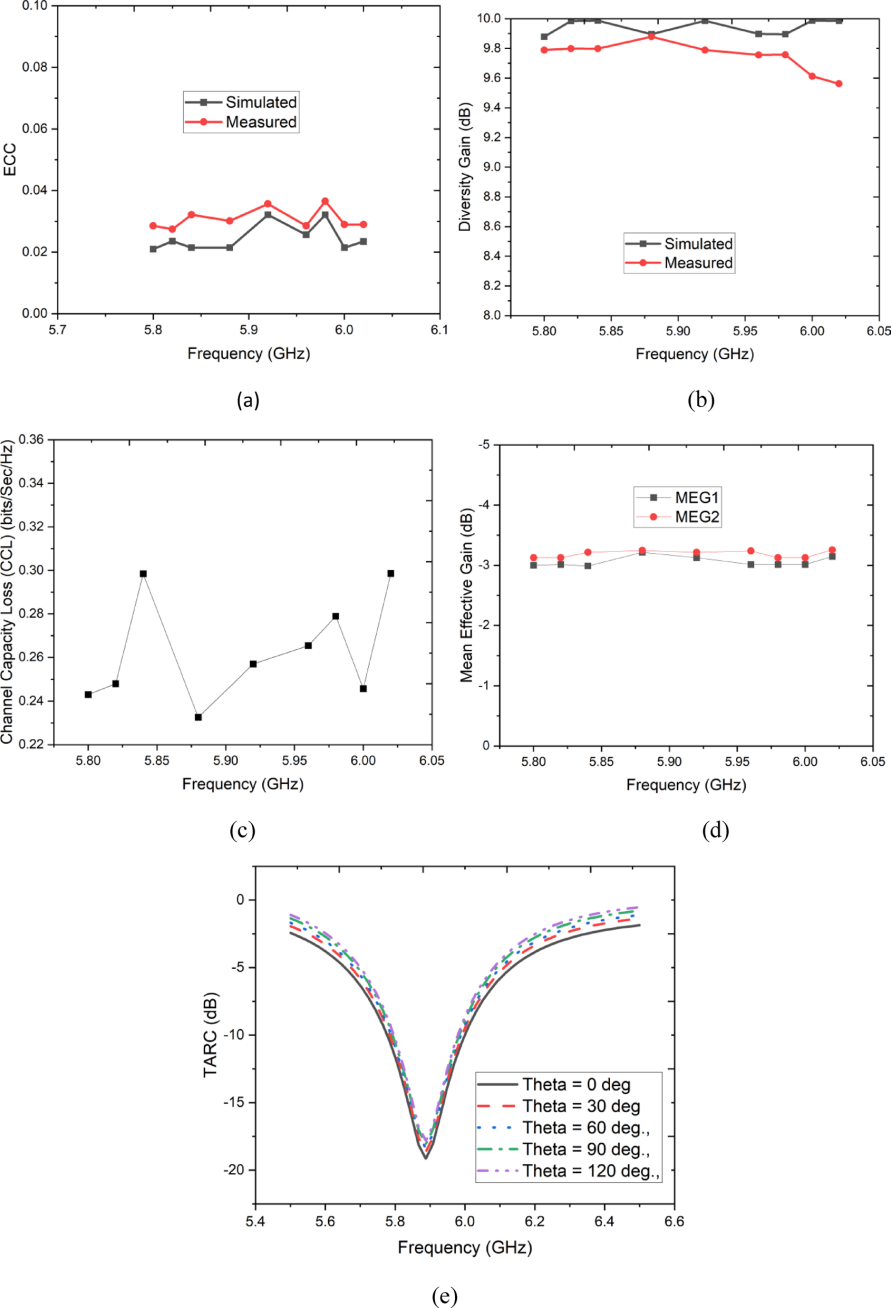
MIMO parameters (**a**) ECC, (**b**) Diversity Gain, (**c**) CCL, (**d**) MEG, (**e**) TARC.

To gain a comprehensive understanding of the Multiple-Input Multiple-Output (MIMO) antenna’s performance, various important parameters are utilized for measurement. Within MIMO systems, the Envelope Correlation Coefficient (ECC) serves to quantify the level of correlation among different antenna ports. Typically, a diversity gain (DG) value exceeding 9.95 dB is essential. The simulated outcomes of ECC and DG are depicted in Fig. 12a,b.

1 $$ECC=\frac{|{{\text{Sii}}^{*}\text{Sij }+{\text{Sji}}^{*}\text{Sjj}|}^{2}}{{(1-|\text{Sii}|}^{2}-{\left|\text{Sij}\right|}^{2}){(1-|\text{Sji}|}^{2}-{\left|\text{Sjj}\right|}^{2})}$$

2 $$Diversity Gain \left(i,j\right)= 10\sqrt{1- {ECC}_{i,j}}$$

Figure 12a indicates a consistent ECC below 0.03 across all frequencies, aligning with the requirement that ECC stays under 0.5. This observation confirms strong isolation between the two ports of the planned MIMO antenna. Additionally, the simulated Diversity Gain (DG) reaches a peak of 9.99 dB across the antenna’s operational frequency range, as displayed in Fig. 12b.

Systems with high diversity gain (DG) offer increased isolation between the radiators and their surroundings. To determine the DG of the MIMO antenna, a specific relationship can be utilized.

3 $${\text{Channel Capacity Loss }}\left( {{\text{CCL}}} \right) = { } - {\text{log}}_{{2{ }}} {\text{det}}\left( {\varphi^{{\text{R }}} } \right)$$

4 $${\varphi }^{R}= \left[\begin{array}{ccc}{ECC}_{\text{ii}}& \cdots & {ECC}_{\text{ij}}\\ \vdots & \ddots & \vdots \\ {ECC}_{\text{ji}}& \cdots & {ECC}_{\text{jj}}\end{array}\right]$$

where,

$${ECC}_{ii}=1-\left(\left({{S}_{ii}}^{2}\right)+ \left({{S}_{ij}}^{2}\right)\right)$$

$${ECC}_{ij}=-\left({{S}_{ii}}^{*}{S}_{ij}+{{S}_{ji}}^{*}{S}_{jj}\right)\text{ for i},\text{ j}=\text{ 1,2}$$

A smaller ECC corresponds to a larger diversity gain, underscoring the superior diversity performance offered by the proposed two-port antenna array. Delving into a broader concept, we examine the MIMO antenna’s channel capacity loss, referred to as CLL. This critical characteristic of data transmission rates, often denoted as CLL, can be calculated using formulas (1) and (2). The impact of the proposed antenna on Channel Capacity reduction is depicted in Fig. 12c. Maintaining an adequate Channel Capacity necessitates keeping the CLL below 0.3 for the entire bandwidth and 0.379 bits/sec/Hz within the specified resonant band. This criterion ensures compliance with the IEEE 802.16 standard and guarantees sufficient data transmission rates for the wide-band MIMO antenna. Furthermore, both the MEG and TARC have been calculated and fall within acceptable ranges, contributing to improved MIMO performance as illustrated in Fig. 12d,e, respectively.

The proposed antenna design demonstrates significant improvements over existing works in terms of compact size, operating frequency, gain, and application suitability as shown in Table 2. With dimensions of 15.22 × 18.55 × 1.6 mm^3^, it is much smaller than most previous designs, such as^25^ (98.1 × 60.4 × 15.7 mm^3^) and^32^ (250 × 250 × 8 mm^3^), making it ideal for space-constrained applications. Operating at 5.9 GHz, the proposed antenna is optimized for V2X communication, a crucial feature lacking in earlier designs focused on GSM, ISM, GPS, or WiMAX. Additionally, it achieves a high gain of 4.5–5.1 dBi, outperforming antennas in^29^ (2.0 dBi),^30^ (2.7 dBi), and^35^ (4.09 dBi), ensuring superior signal transmission and reception. Unlike some prior works that use costly materials like RO4003 and Rogers 5880, this antenna utilizes FR4, a cost-effective and widely available dielectric, making it practical for mass production. Given these advantages, the proposed antenna is a highly efficient and affordable solution for modern V2X communication systems, providing enhanced performance while maintaining low fabrication costs.

**Table 2:** Performance comparison of the proposed antenna with literature.

References	Antenna size (mm^3^)	Operating frequency (GHz)	Gain (dBi)	Dielectric used	Application
^25^	98.1 × 60.4 × 15.7	0.790–2.060	–	RO4003	GSM
^26^	60 × 60 × -	1.575	6.9	RT/duroid 5870	–
^27^	50 × 50 × 1.524 (3 × 3 array)	5.13–6.24	9.8	Rogers 5880	ISM
^28^	40 × 40 × 1.6	2.45	5.32	FR4	ISM
^29^	80 × 55 × 1.6	1.6	2.0	FR4	GPS
^30^	150 × 150 × 1.6	3.06	2.7	FR4	–
^31^	34 × 34 × 1.6	1.8/3.5/5.2/5.8	1.7–4.4	FR4	GSM/ISM
^32^	250 × 250 × 8	0.430	4.36	EVA Foam	–
^33^	50 × 50 × 0.8	2.45/4.27	3.17/3.83	FR4	ISM
^34^	26 × 25 × 0.2	1.575/2.45/5.2	3.6–4.04	Rogers RO4003	ISM
^35^	50 × 55 × 1.6	2.21/3.42/4.85/8.02/10.19	4.09	FR4	4G, 5G, and X
^36^	40 × 40 × 2.5	1.6/2.4/2.5	1.96–5.2	Rogers 6010LM	L1/WiMAX/Sat Com
^37^	32 × 98 × 1	0.6/1.8/2.4/3.5/5.5	5.14	FR4	GSM/ISM/WiMAX
^38^	62 × 58 × 10	4.27/5.8	5.0–7.1	EVA foam	C and ISM
Proposed work	15.22 × 18.55 × 1.6	5.9	4.5–5.1	FR4	V2X

### SAR analysis

**Fig. 13:**
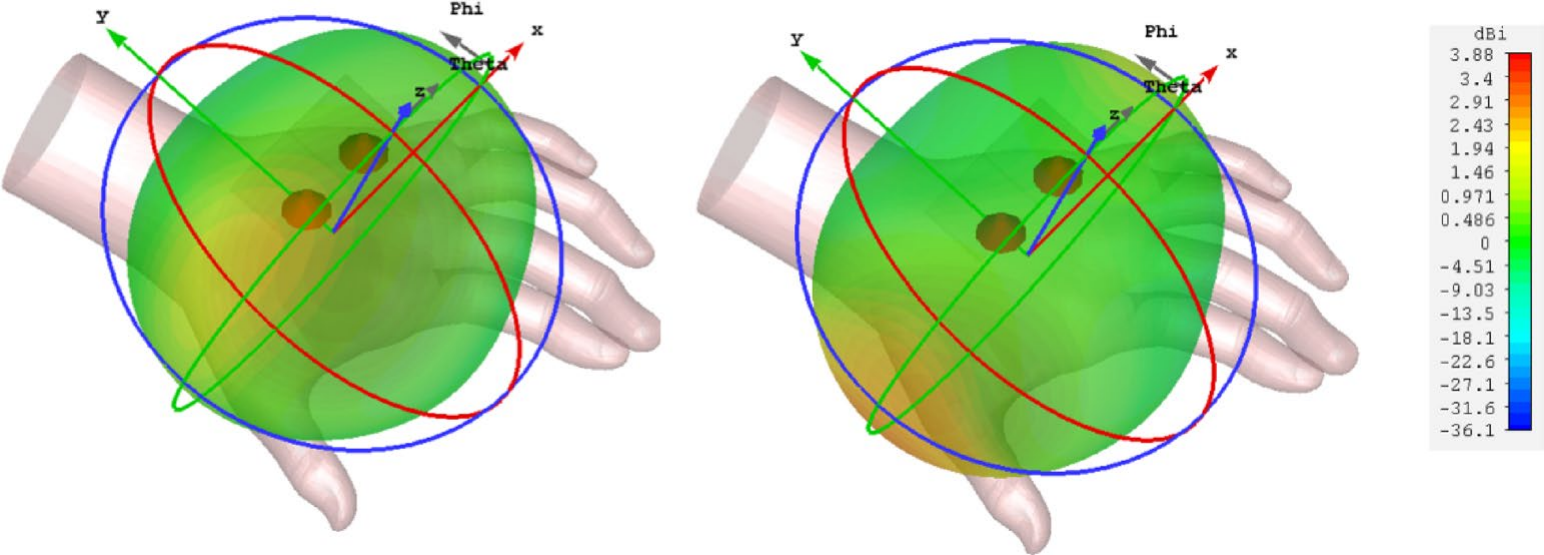
Simulated 3D radiation pattern of the proposed antenna when placed on the human hand model.

**Fig. 14:**
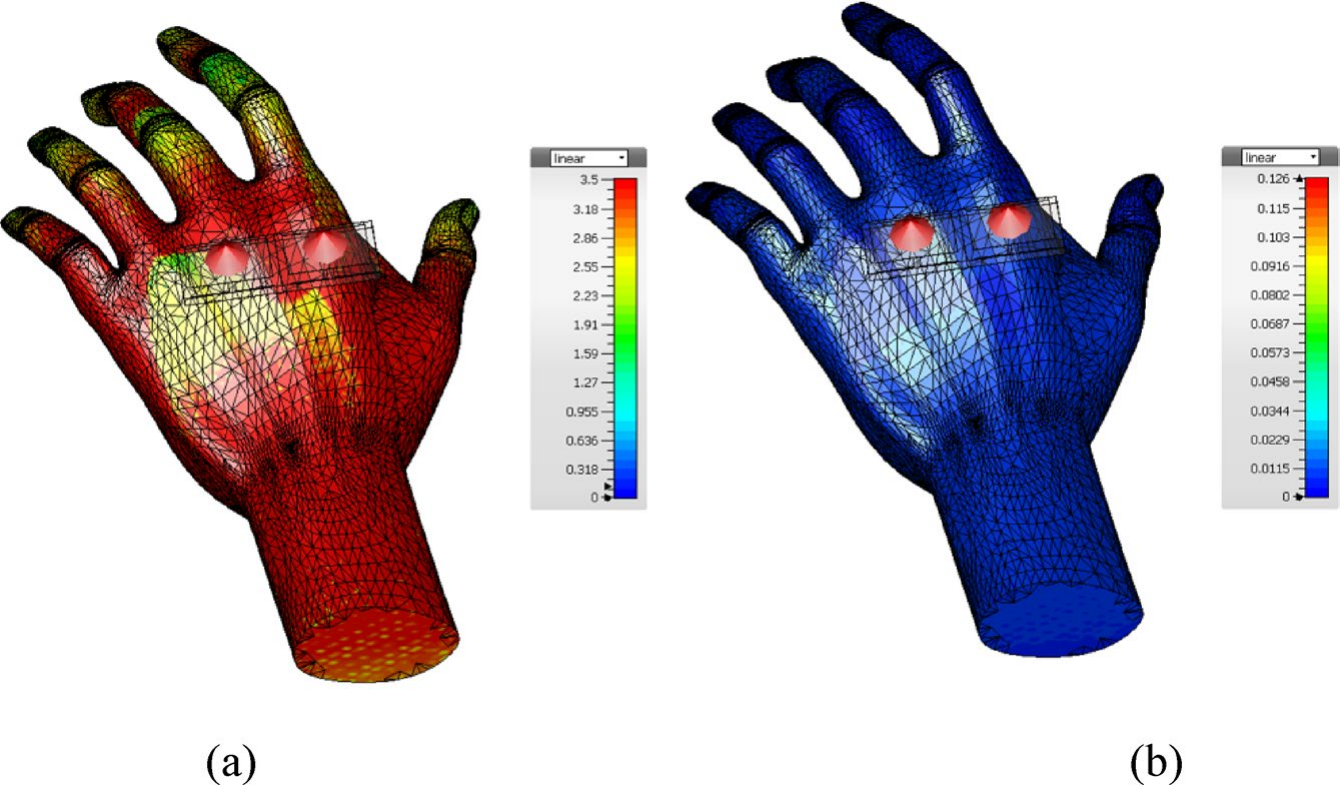
SAR analysis of the proposed antenna (**a**) Conventional Patch antenna (**b**) Proposed antenna.

The designed antenna performance was validated after placing the human hand phantom model, and a corresponding three-dimensional far-field pattern was obtained for both elements, as shown in Fig. 13a,b. It is well observed that the antenna radiates all its energy above the hand phantom and none coming towards the hand, ensuring that skin tissues have not been affected by electromagnetic radiation. Figure 14 shows the SAR analysis of the proposed antenna.

## Conclusion

Based on the findings presented in the research article, the hybrid approach utilizing the rectangular strip and connecting strip methods stands out as a promising solution to address the challenge of significant back lobe radiation in high front-to-back ratio (FBR) microstrip patch antennas. This innovative technique effectively counters backward radiation by leveraging supplementary magnetic currents induced through an inductive effect. The resulting reduction in backward radiation from the original patch showcases the efficiency of this single antenna element, which occupies a notably smaller area, making it highly suitable for wearable applications, with dimensions approximately 0.029 λ × 0.036 λ at the 5.9 GHz cutoff wavelength. Moreover, the comprehensive insights provided by the equivalent circuit model elucidate the coordinated action, offering a deeper understanding of the underlying physical mechanisms driving the observed outcomes. The achieved FBR surpassing directivity by up to 6.03 dB not only enhances performance but also leads to a diminished SAR when worn. Importantly, this improvement in FBR is achieved while maintaining minimal impact on radiation efficiency in the presence of the human body, a crucial quality for wearable devices. In summary, this research introduces a viable and efficient method to mitigate back lobe radiation in microstrip patch antennas, paving the way for enhanced performance and reduced SAR in wearable applications. The findings presented here contribute valuable insights to the field and offer a practical solution to a significant challenge in antenna design.

## Data Availability

The data used to support the findings of this study are included in the article. Data will be provided on request from Rajesh Kumar D, Email: sdrk87@gmail.com and S. Sesha Vidhya,Email:seshavidhya@rmkcet.ac.in.
